# Bioactive phytochemicals, pharmacological, and therapeutic potential of *Dillenia indica*: A comprehensive review of current research

**DOI:** 10.1016/j.chmed.2025.09.001

**Published:** 2025-09-04

**Authors:** Lutfun Nahar, Emran Habibi, Chuanchom Khuniad, Kulyash Kalieva, Daijie Wang, Hesamoddin Arabnozari, Phanuphong Chaiwut, Sarita Sangthong, Tinnakorn Theansungnoen, Rajat Nath, Anupam Das Talukdar, Satyajit D. Sarker

**Affiliations:** a Laboratory of Growth Regulators, Palacký University and Institute of Experimental Botany, the Czech Academy of Sciences, Šlechtitelů 27, Olomouc 77900, Czech Republic; b Medicinal Plants Research Centre, Institute of Herbal Medicines and Metabolic Disorders, Mazandaran University of Medical Sciences, Sari 48175-866, Iran; c Centre for Natural Products Discovery, School of Pharmacy and Biomolecular Sciences, Liverpool John Moores University, Liverpool L3 3AF, United Kingdom; d Department of Thai Traditional Medicine, Faculty of Health and Sports Science, Thaksin University, Phatthalung 93210, Thailand; e Deapartment of Chemistry & Mathematics, Al-Farabi Kazakh National University, Almaty 050040, Republic of Kazakhstan; f International Joint Laboratory of Medicinal Food Development and Health Products Creation, Biological Engineering Technology Innovation Center of Shandong Province, Heze Branch of Qilu University of Technology (Shandong Academy of Sciences), Heze 274015, China; g School of Cosmetic Science, Mae Fah Luang University, Chiang Rai 57100, Thailand; h Department of Biotechnology and Microbiology, School of Natural Sciences, Techno India University, Agartala 799004, India; i Department of Life Science and Bioinformatics, Assam University, Assam 788011, India

**Keywords:** anticancer activity, antioxidant potential, bioactive phytochemicals, *Dillenia indica* L., pharmacological properties, preclinical and clinical studies

## Abstract

*Dillenia indica*, commonly known as “elephant apple”, is a tropical fruit-bearing tree with a rich history of medicinal use in traditional medicine across Southeast Asia and the Indian subcontinent. This review comprehensively examines current research on the pharmacological properties of *D. indica*, focusing on its diverse bioactive compounds, including flavonoids, tannins, saponins, and triterpenoids, which underpin its key biological activities such as antioxidant, anti-inflammatory, antimicrobial, anticancer, antidiabetic, and hepatoprotective properties. Flavonoids and tannins exhibit potent antioxidant activity, effectively scavenging free radicals and reducing oxidative stress; a mechanism implicated in metabolic disorders, cancer, and neurodegenerative diseases. Additionally, the plant demonstrates considerable anti-inflammatory effects by modulating key inflammatory pathways, including the inhibition of pro-inflammatory cytokines such as tumor necrosis factor-*α* (TNF-*α*) and interleukin-6 (IL-6). Saponins and triterpenoids enhance its antimicrobial activity, supporting traditional uses for treating infections. Preclinical studies indicate that *D. indica* extracts can inhibit cancer cell proliferation and induce apoptosis in various cancer types, including breast, colorectal, and liver cancers. Furthermore, specific formulations may improve the bioavailability and targeted delivery of active constituents, thereby increasing therapeutic efficacy. Despite the promising findings *in vitro* and *in vivo*, there is a notable lack of well-designed clinical trials to validate these effects in humans. The plant is generally considered safe when consumed at recommended doses. However, excessive intake may lead to gastrointestinal discomfort and, in rare cases, hepatotoxicity. This review describes the therapeutic potential of *D. indica* as a natural agent while emphasizing the urgent need for comprehensive clinical trials, particularly those investigating pharmacodynamics profiles, to confirm its efficacy and safety.

## 1 Introduction

*Dillenia indica* L., widely known as “elephant apple” or “chalta”, is a tropical evergreen tree native to Southeast Asia and the Indian subcontinent (Kumar, Kumar, & Prakash, 2011). It thrives in humid climates with abundant rainfall, typically growing near riverbanks and in lowland forests (Rai & Sajwan, 2020). The plant typically grows near riverbanks and in lowland forests, where conditions favour its growth (Nagar et al., 2020, Dasanayaka et al., 2022). This species holds significant value in traditional medicinal practices throughout Southeast Asia. Various parts of the plant, including the fruit, leaves, and bark, are utilized in traditional medicinal systems, such as Ayurvedic and Unani practices, to treat a wide range of ailments (Alam et al., 2020, Boruah et al., 2020, Rai and Sajwan, 2020, Rizzo et al., 2020). Various parts of the plant, such as fruit, leaves, bark and roots, have been traditionally used to treat diverse health conditions (Sabandar, Jalil, Ahmat, & Aladdin, 2017).

The pharmacological potential of *D. indica* has attracted considerable attention, largely due to its rich composition of bioactive compounds (Singh & Saha, 2019). These compounds include flavonoids, tannins, saponins, and phenolic acids (Sabandar, Jalil, Ahmat, & Aladdin, 2017), which contribute to its antioxidant, anti-inflammatory, antimicrobial, and anticancer activities (Yeshwante et al., 2009, Kaur et al., 2018). Flavonoids and phenolic acids are known for their ability to neutralize free radicals and reduce oxidative stress, a factor linked to chronic diseases (Alam et al., 2020, Sahariah et al., 2023). Saponins and tannins further modulate immune responses and exhibit antimicrobial properties, thereby enhancing the medicinal significance of this species (Padmavathi et al., 2011, Boparai et al., 2016).

Despite its extensive traditional use, *D. indica* remains underutilized in modern medicine (Kamboj, Talukdar, & Banerjee, 2019). There is a critical need for further research to establish standardized formulations and validate its clinical efficacy. Recent studies have explored both fruit-derived polysaccharides and bark-mediated nanoparticle formulations, thus offering new therapeutic avenues (Mohanta and Jena, 2023, Mohanta et al., 2024). Specifically, the bark extract has been used to fabricate copper nanoparticles with potent antioxidant and anticancer activity, while fruit-derived polysaccharides exhibit strong radical scavenging capacity, biocompatibility, and *in vivo* safety (Mohanta and Jena, 2023, Mohanta et al., 2024). These findings highlight the pharmacological versatility of *D. indica* and support its potential for safe and effective therapeutic development. This review provides a comprehensive evaluation of *D. indica*, with an emphasis on its pharmacological significance and therapeutic potential in both traditional and modern contexts.

Preclinical studies highlight its efficacy in managing oxidative stress, inflammation, microbial infections, and cancer; however, clinical validation remains limited (Aswathy et al., 2021, Lawal et al., 2021). The integration of traditional medicinal knowledge with modern pharmacological research underscores the necessity for further scientific investigations. This approach may position *D. indica* as a promising candidate for future therapeutic innovations.

## 2 Botanical description and ecological importance

*D. indica* belongs to the Dilleniaceae family and the order Dilleniales. Commonly known as elephant apple, this plant is recognized for its medicinal uses across Asian countries (Gandhi and Mehta, 2014, Sharma et al., 2023). The tree can grow up to 20 m in height and features large, spirally arranged, leathery leaves that can measure up to 30 cm in length (Kumar et al., 2011, Rizzo et al., 2020). The leaves have serrated edges and a deeply veined pattern, which enhance photosynthetic efficiency by maximizing light capture and improving water drainage (Saiful Yazan & Armania, 2014).

**Fig. 1:**
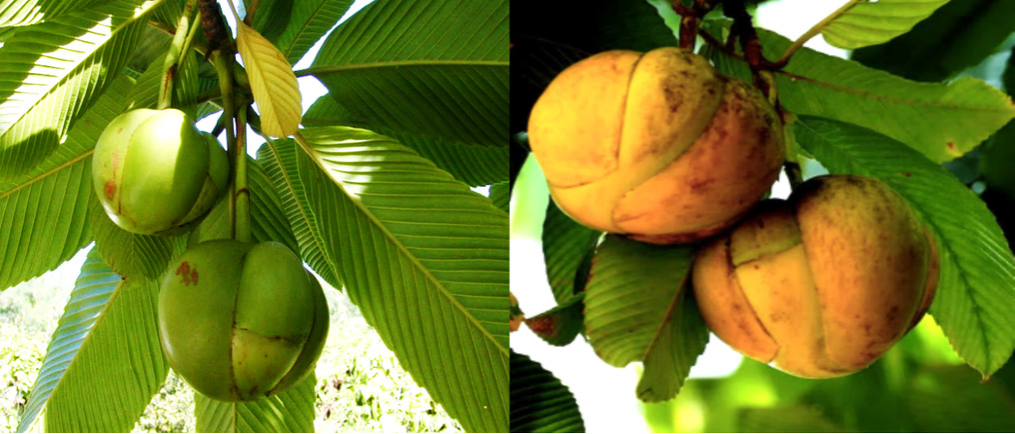
Unripe (left) and ripe fruits (right) of *D. indica* (Elephant apple).

During the rainy season, the plant produces large, fragrant white flowers that attract pollinators, including bees and birds, thereby aiding in its reproductive cycle (Yeshwante et al., 2009, Nagar et al., 2020, Petit et al., 2024). The fruit of *D. indica* is large, spherical, and has a tough, rough exterior. It ripens from green to a distinctive yellow (Fig. 1) and contains a fibrous pulp that is sour and rich in seeds (Gogoi et al., 2020, Kumar et al., 2023).

Ecologically, *D. indica* plays a crucial role in maintaining biodiversity within its native tropical rainforests and lowland ecosystems (Saiful Yazan and Armania, 2014, Kamboj et al., 2019). The tree thrives in diverse soil types, ranging from acidic to neutral, and is commonly found near freshwater sources (Rai & Sajwan, 2020). The plant serves as a food source for various animals, particularly elephants, which contribute to its seed dispersal and propagation, reinforcing its common name, “elephant apple” (Alam et al., 2012, Kumar et al., 2023). Birds, bats, and other wildlife also consume the fruit, assisting in its distribution across the region (Sen et al., 2018, Mondal and Mani, 2021, Mohanta and Jena, 2023).

In addition to being a food source, *D. indica* plays a critical role in maintaining ecosystem stability (Nagar, Chourasia, Upadhyay, Gururani, & Rathi, 2020). The tree provides shade, reduces soil erosion, and supports local biodiversity. Its flowers and leaves nourish various insects, which are integral to sustaining local food webs (Pattanayak, 2023). This botanical and ecological overview emphasizes the multifaceted value of *D. indica*, highlighting its significant potential for ecological conservation and therapeutic development stemming from its environmental contributions and applications in traditional medicine.

## 3 Ethnobotanical significance and traditional uses

**Fig. 2:**
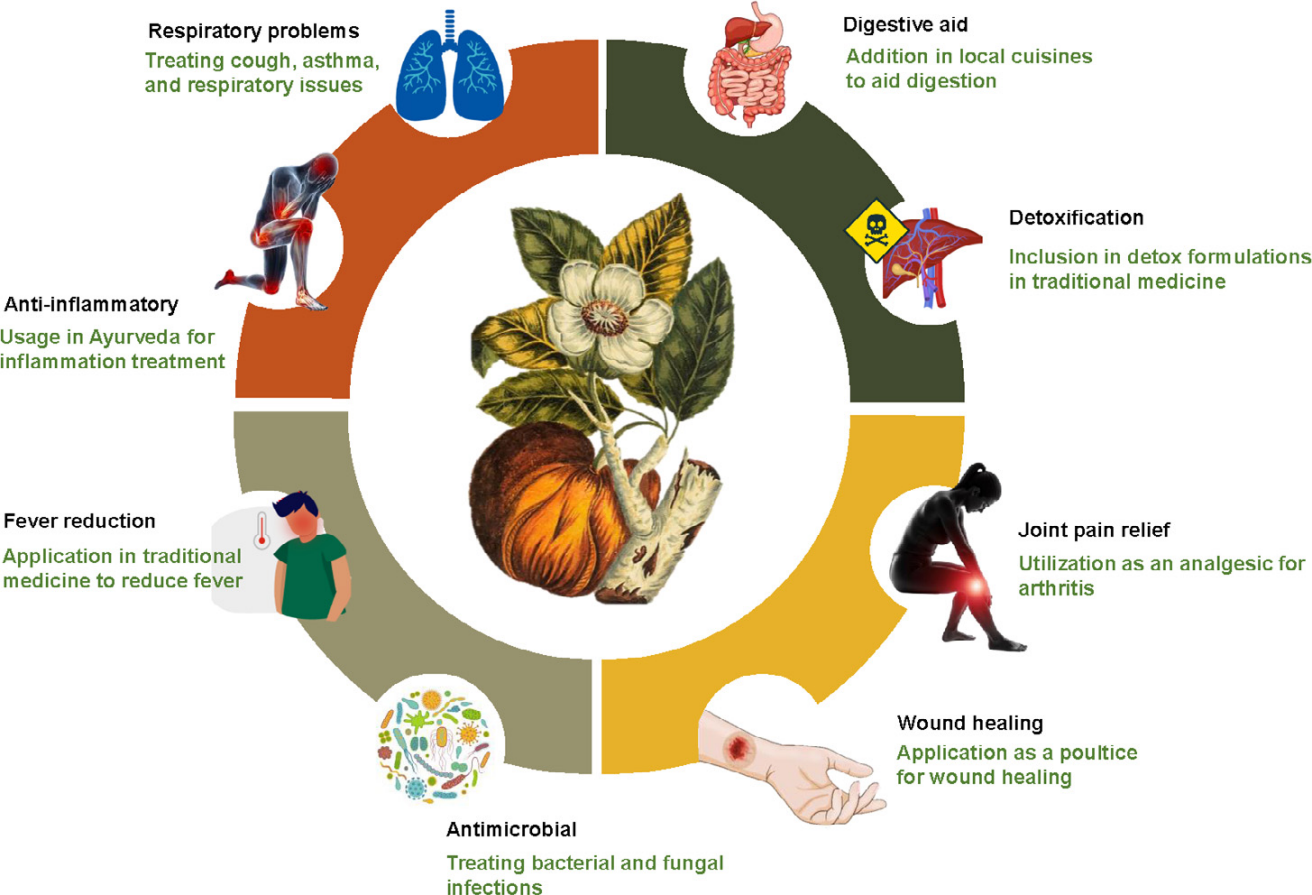
Summary of ethnobotanical uses of *D. indica*.

*D. indica* has been an integral part of traditional medicine across South and Southeast Asia, particularly in Bangladesh, India, Sri Lanka, and Thailand. Its ethnobotanical significance is rooted in generations of folk remedies passed down through cultural practices (Sahu et al., 2011, Alam et al., 2012, Talukdar et al., 2012, Boruah et al., 2020, Song et al., 2022). Modern scientific research increasingly validates these traditional applications, highlighting the pharmacological potential of this species. Various parts of the plant, including the fruit, bark, leaves, and roots, are utilized to treat a wide range of ailments. A summary of the ethnobotanical uses of *D. indica* is shown in Fig. 2. The continued relevance of *D. indica* in modern herbal medicine reflects not only its rich medicinal history but also its adaptability to contemporary health needs. Traditional knowledge serves as a valuable foundation for modern pharmacological studies that aim to uncovering the full therapeutic benefits of this plant (Talukdar, Talukdar, Deka, & Sahariah, 2012).

Traditionally, people have consumed the fruit of *D. indica* to aid digestion (Nagar, Chourasia, Upadhyay, Gururani, & Rathi, 2020) and utilized various parts for detoxification to cleanse the body (Gogoi, Sahariah, Patowary, Mawlieh, Deka, & Bordoloi, 2020). The plant is recognized for its ability to alleviate fever (Padmavathi, Deshpande, & Sarala, 2011), while the bark and leaves are applied as poultices for wound healing (Boparai, Niazi, Bajwa, & Singh, 2016). In Ayurveda, *D. indica* is noted for its anti-inflammatory properties (Padmavathi, Deshpande, & Sarala, 2011). Additionally, its plant parts are employed to treat respiratory issues, including cough and asthma (Kaur, Kishore, & Singh, 2018). The bark is commonly used as an analgesic for joint pain and arthritis (Alam et al., 2012, Song et al., 2022). Both the bark and leaves exhibit significant antimicrobial properties, making them effective in treating bacterial and fungal infections (Biswas and Pandita, 2015, Mohanta and Jena, 2023). This broad spectrum of traditional uses illustrates the rich phytochemical profile of *D. indica*, highlighting its diverse therapeutic potential.

Scientific studies support these traditional applications, confirming the anti-inflammatory, antimicrobial, and antioxidant properties of *D. indica* (Boparai et al., 2016, Kaur et al., 2018). Research has shown that its bioactive compounds inhibit enzymes linked to inflammation and neutralize free radicals, thereby reinforcing its therapeutic potential (Alam et al., 2020, Sahariah et al., 2023). The plant is being explored for its role in managing metabolic disorders, including diabetes (Talukdar et al., 2012, Kamboj et al., 2019). Recent advancements have led to the development of novel therapeutic formulations, such as nasal gels incorporating *Dillenia* mucilage for enhanced medicinal applications (Sahu et al., 2011, Mohanta and Jena, 2023). The integration of traditional knowledge with modern scientific research highlights the potential of *D. indica* in developing natural therapeutics for managing conditions such as diabetes, cancer, and infections (Aswathy et al., 2021, Lawal et al., 2021).

## 4 Bioactive phytochemicals in *D. indica*

*D. indica* is a rich source of bioactive phytochemicals that contribute to its medicinal properties. Various parts of the plant, including the fruit, bark, leaves, and seeds, contain diverse bioactive phytochemicals such as flavonoids, tannins, anthocyanidins, saponins, triterpenoids, phenolic acids, alkaloids, polysaccharides, and glycosides (Kumar et al., 2011, Padmavathi et al., 2011, Sabandar et al., 2017). These compounds exhibit significant pharmacological activities, including antioxidant, anti-inflammatory, antimicrobial, anticancer, and antidiabetic activities (Yeshwante et al., 2009, Boparai et al., 2016).

An overview of the major phytochemicals found in *D. indica,* and their therapeutic significance is shown in Table 1. The presence of these bioactive phytochemicals reflects the potential of *D. indica* to serve as a natural remedy for various ailments, supporting its historical use in traditional medicine. The diverse chemical profile further supports its importance in both dietary and therapeutic applications.

**Table 1:** Major bioactive compounds in *D. indica* and their therapeutic effects.

Phytochemical class	Identified compounds	Source	Therapeutic effects	References
Flavonoids	Quercetin; Kaempferol; Myricetin; Dillenetin; Rhamnetin; Isorhamnetin; Kaempferide; Kaempferide 3-*O*-di-glucoside; Dihydrokaempferide; Dihydrokaempferide 7-di-glucoside; 4,5,7,30,40-Pentahydroxy flavan-3-*O*-*β*-*D*-glucopyranoside; Leucocyanidin; Naringenin; 3,5,7-Trihydroxy-2-(4-hydroxy-benzyl)-chroman-4-one	Leaves, bark, fruit	Antioxidant, anti-inflammatory, anticancer	Kaur et al., 2018, Alam et al., 2020, Saikia et al., 2023
Tannins	Gallotannins; Ellagitannins	Fruit, bark	Antioxidant, antimicrobial, astringent	Padmavathi et al., 2011, Boparai et al., 2016
Anthocyanidins	Proanthocyanidins (B-type); Prodelphinidins (B-type)	Fruit	Antioxidant, antimicrobial	Fu, Yang, Peh, Lai, Feng, & Yang, 2015
Saponins	Diosgenin; other saponins	Leaves, bark	Antimicrobial, immune boosting	Yeshwante et al., 2009, Sabandar et al., 2017
Triterpenoids	Lupeol; Betulin; Betulinaldehyde; Betulinic acid; 3*β*-Hydroxylupane-13*β*,28-lactone	Bark, leaves	Anti-inflammatory, anticancer	Boparai et al., 2016, Singh and Saha, 2019
Phenolic acids	Gallic acid; Ellagic acid	Fruit, leaves	Antioxidant, anti-inflammatory	Kaur et al., 2018, Alam et al., 2020
Alkaloids	Berberine; Colchicine	Various parts	Analgesic, antimicrobial	Alam et al., 2012, Kamboj et al., 2019
Polysaccharides	Pectin; other polysaccharides	Fruit	Antioxidant, immunomodulatory	Mohanta & Jena, 2023
Miscellaneous compounds	Amino butyric acid; Glutamic acid; *n*-Hentriacontanol; *n*-Heptacosan-7-one; *n-*Honatriacontan-18-one; 1,8-Dihydroxy-2-methyl anthraquinone-3-*O*-*β*-*D*-glucopyranoside	Fruit	Antioxidant	Sabandar, Jalil, Ahmat, & Aladdin, 2017

### 4.1 Flavonoids

**Fig. 3:**
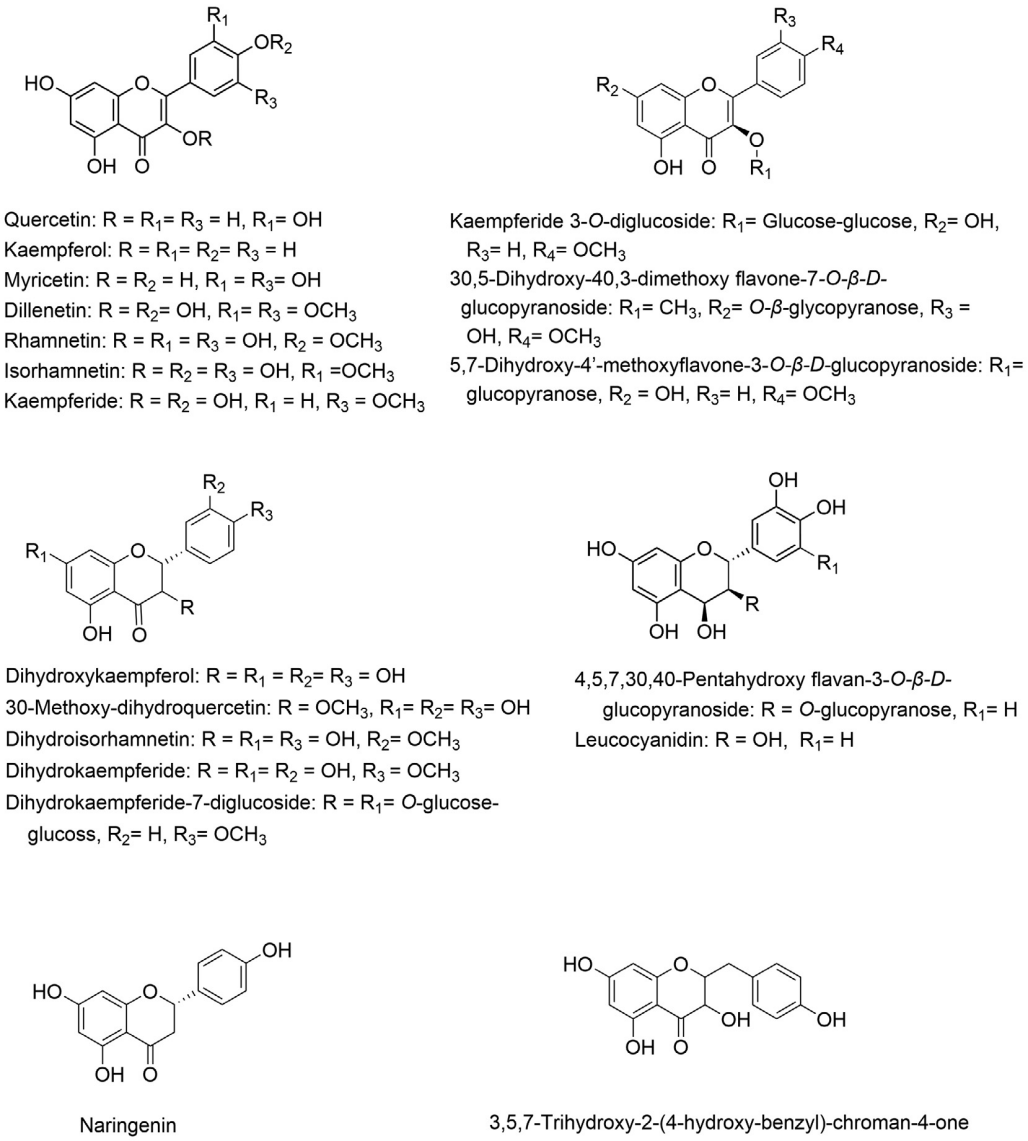
Major flavonoids from *D. indica*.

*D. indica* contains a diverse range of flavonoids, including quercetin, kaempferol, myricetin, and several other flavonoids as illustrated in Table 1 and Fig. 3. These compounds exhibit strong antioxidant and anti-inflammatory properties as demonstrated by *in vitro* 1,1-diphenyl-2-picrylhydrazyl (DPPH) and 2,2′-azino-bis (3-ethylbenzothiazoline-6-sulfonic acid) (ABTS) assays and the inhibition of pro-inflammatory cytokines in RAW 264.7 cells, thereby helping to reduce oxidative stress, prevent chronic diseases and support cardiovascular health (Kaur et al., 2018, Alam et al., 2020).

Quercetin has been shown to scavenge free radicals and inhibit pro-inflammatory cytokines (Saikia, Kesavan, Stephen Inbaraj, Dikkala, Nayak, & Sridhar, 2023). Kaempferol enhances cardiovascular health by preventing the oxidation of low-density lipoproteins (LDL), thereby reducing the risk of atherosclerosis. Additionally, its anticancer properties have been demonstrated by its ability to induce apoptosis in cancer cells while inhibiting their proliferation (Kamboj et al., 2019, Singh and Saha, 2019). Myricetin complements these effects by offering additional anti-inflammatory benefits and inhibiting pro-inflammatory mediators such as nitric oxide and various cytokines, a property that is advantageous for managing chronic inflammatory conditions (Kaur et al., 2018, Alam et al., 2020).

### 4.2 Tannins and proanthocyanidins

**Fig. 4:**
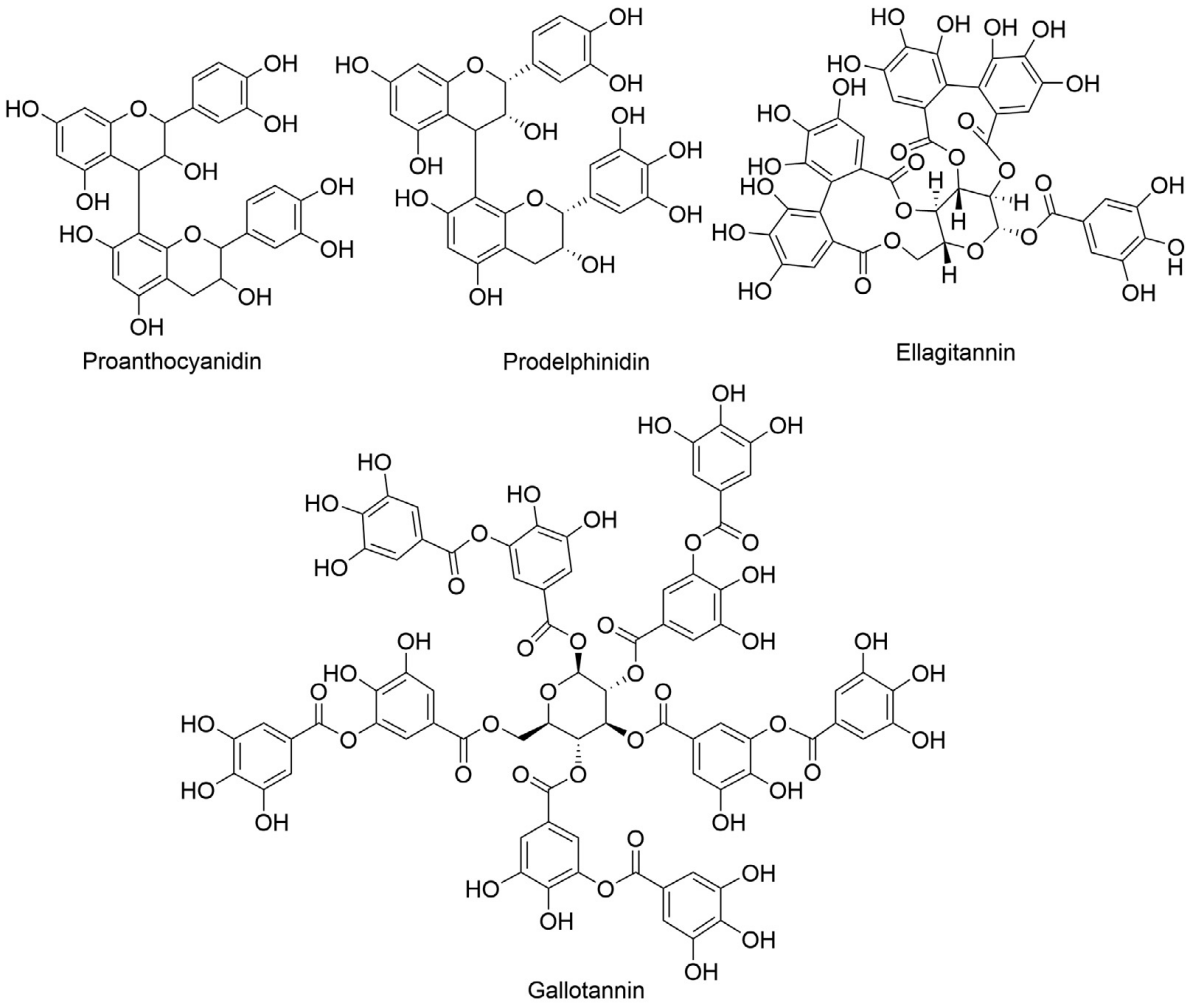
Isolated tannins (gallotannins, ellagitannins) and proanthocyanidins (procyanidins and prodelphinidins) of *D. indica.*

They have powerful antimicrobial and anti-inflammatory properties and may aid in wound healing and digestive health. The fruits and bark of *D. indica* contain tannins, which contribute to various therapeutic effects. Tannins are broadly classified into two categories: hydrolysable and condensed tannins. Hydrolysable tannins (Fig. 4), such as gallotannins and ellagitannins, are important for gastrointestinal health, assisting in the management of gastrointestinal disorders (Padmavathi et al., 2011, Boparai et al., 2016).

On the other hand, condensed tannins (Fig. 4), also known as proanthocyanidins, are oligomeric and polymeric flavan-3-ol compounds commonly found in the plant kingdom (Wallace & Giusti, 2010). Research has revealed that the proanthocyanidins in *D. indica* fruit primarily consist of B-type procyanidins, with a minor amount of B-type prodelphinidins (Fu, Yang, Peh, Lai, Feng, & Yang, 2015). These condensed tannins enhance antimicrobial activity against bacterial pathogens such as *Staphylococcus aureus* and *E. coli* (Yeshwante et al., 2009).

### 4.3 Saponins

**Fig. 5:**
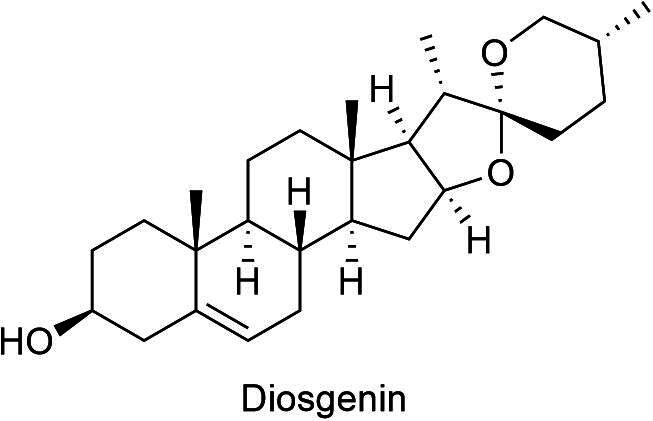
Diosgenin, a bioactive sapogenin from *D. indica.*

These compounds are highly abundant in the leaves and bark of *D. indica* and are recognized for their immune-modulating and antimicrobial activities. Saponins such as those in the plant, exhibit surfactant properties that disrupt microbial cell membranes, contributing to their antibacterial, antifungal, and antiviral potential (Kamboj et al., 2019, Rai and Sajwan, 2020, Rizzo et al., 2020). In addition to their antimicrobial properties, saponins also modulate immune responses. Diosgenin (Fig. 5), a specific sapogenin found in *D. indica*, is linked to cholesterol-lowering effects and glucose metabolism regulation, which may aid in managing diabetes (Mohanta & Jena, 2023). Overall, the presence of saponins in *D. indica* contributes to its diverse pharmacological profile, reinforcing its traditional applications in herbal medicine.

### 4.4 Triterpenoids

**Fig. 6:**
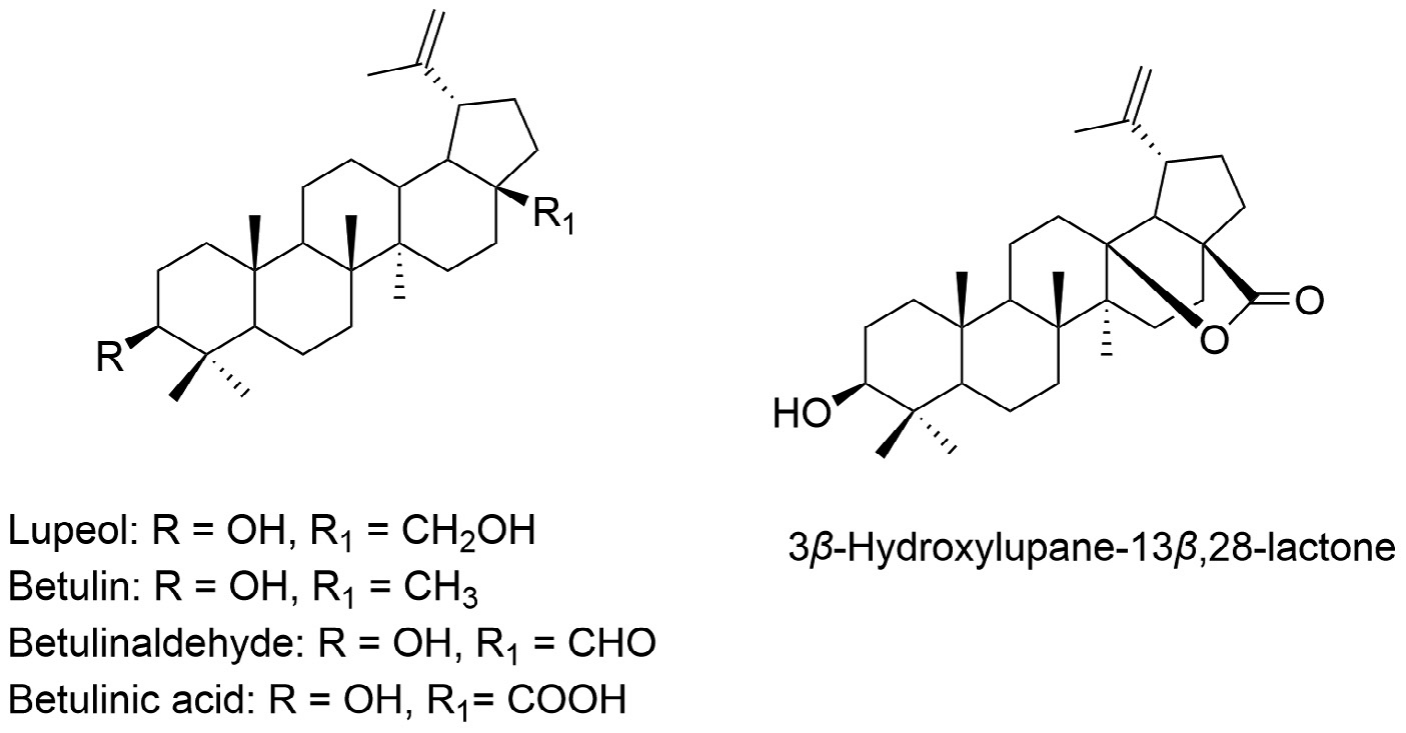
Lupeol, betulin, betulinaldehyde, betulinic acid, and 3*β*-hydroxylupane-13*β* bioactive triterpenes from *D. indica.*

Compounds such as betulinic acid and lupeol (Fig. 6) are well-known triterpenoids present in *D. indica*. These compounds are notable for their significant anti-inflammatory and anticancer activities (Singh & Saha, 2019). Betulinic acid is particularly recognized for its ability to induce apoptosis in cancer cells, showing promise as a therapeutic agent against melanoma and breast cancer (Kaur et al., 2018, Saikia et al., 2023). Lupeol also contributes to the anti-inflammatory benefits of *D. indica* by inhibiting enzymes like cyclooxygenase-2 (COX-2) and lipoxygenase (LOX). This action helps reduce inflammation associated with conditions such as arthritis and cardiovascular diseases (Lawal, Raut, Patel, & Mahady, 2021). The diverse effects of triterpenoids highlight their role in mitigating inflammation and providing protective effects through various biochemical pathways. This positions *D. indica* as a valuable source of triterpenoids for potential use in natural therapeutic applications.

### 4.5 Phenolic acids

**Fig. 7:**
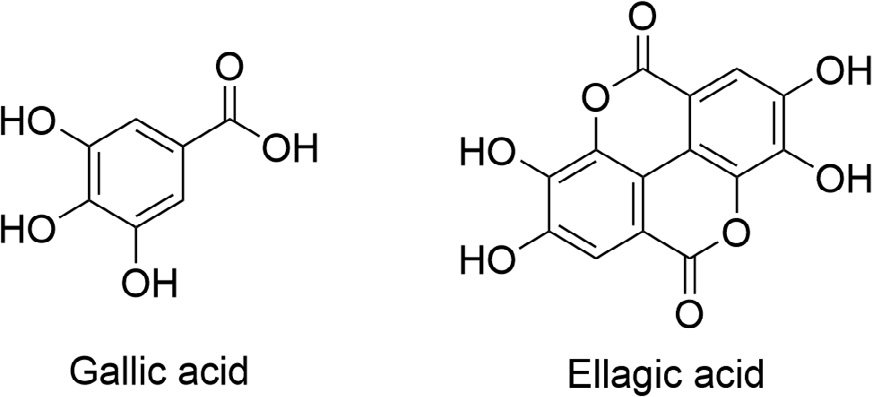
Gallic acid and ellagic acid, two phenolic acids from *D. indica.*

*D. indica* is rich in phenolic acids such as gallic acid and ellagic acid (Fig. 7), which are particularly concentrated in the fruit and leaves (Kaur et al., 2018, Alam et al., 2020). These compounds are known for their strong antioxidant and anti-inflammatory properties. Gallic acid effectively suppresses the production of pro-inflammatory cytokines, making it valuable for addressing chronic inflammatory conditions and cardiovascular diseases (Kaur et al., 2018, Alam et al., 2020). Similarly, ellagic acid offers protective benefits against oxidative stress and DNA damage. This compound contributes to the plant’s anticancer potential by preventing cellular mutations that could lead to tumour development (Saikia, Kesavan, Stephen Inbaraj, Dikkala, Nayak, & Sridhar, 2023). The presence of these phenolic acids supports the therapeutic reputation of *D. indica* and aligns well with its traditional applications for various health conditions.

### 4.6 Alkaloids

While *D. indica* contains alkaloids in smaller quantities, these compounds play important pharmacological roles, such as analgesic and anti-inflammatory actions. Alkaloids are known for their diverse effects on the human body, particularly through interactions with various receptors in the nervous system. This interaction facilitates effective pain relief while reducing inflammation (Alam et al., 2012, Kamboj et al., 2019, Rai and Sajwan, 2020, Rizzo et al., 2020).

**Fig. 8:**
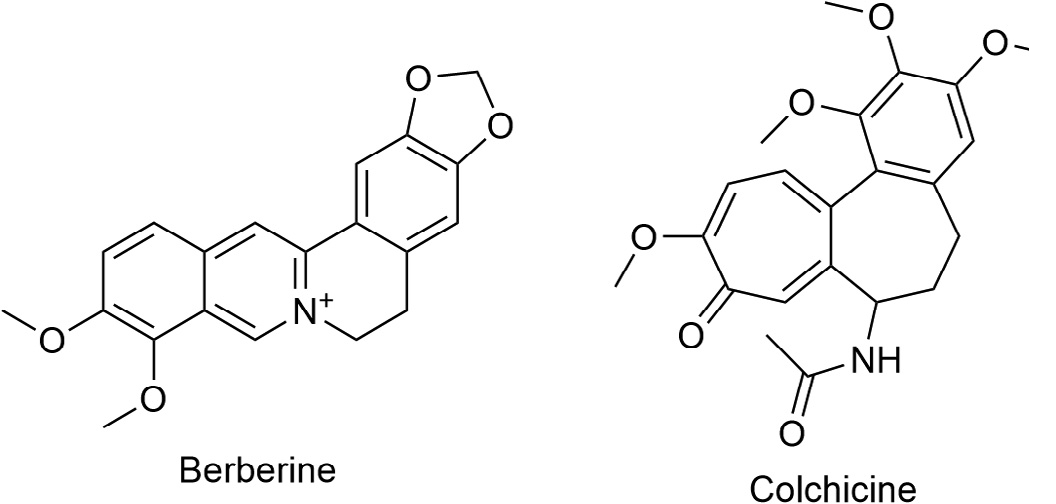
Berberine and colchicine, two bioactive alkaloids from *D. indica.*

Berberine (Fig. 8) is one of the notable alkaloids identified in *D. indica*. This compound has garnered attention for its broad-spectrum antimicrobial properties, showing efficacy against several pathogens, including bacteria, viruses, and fungi (Kaur, Kishore, & Singh, 2018). Berberine has demonstrated various biological activities, including antibacterial, antifungal, and antiviral properties, making it a potential resource for developing treatments for infectious diseases. Colchicine (Fig. 8), another alkaloid found in *D. indica*, is recognized for its anti-inflammatory properties. It is particularly beneficial in treating conditions such as gout and familial Mediterranean fever. Colchicine can inhibit the migration of leukocytes to sites of inflammation, thereby reducing the overall inflammatory response (Kamboj, Talukdar, & Banerjee, 2019). Overall, while the alkaloid content in *D. indica* may be less prominent compared to other phytochemicals, their presence adds to the complex pharmacological profile of this species and offers additional avenues for therapeutic development.

### 4.7 Polysaccharides

**Fig. 9:**
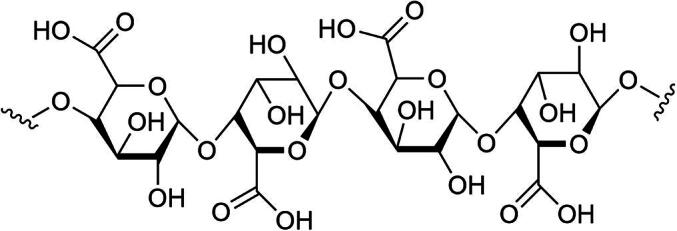
A well-known polysaccharide, pectin.

A well-known polysaccharide, pectin (Fig. 9), was extracted from the fruit of *D. indica*, exhibits significant antioxidant activity essential for neutralizing free radicals responsible for oxidative stress (Kaur et al., 2018, Silva et al., 2024). This high-molecular-weight compound is effective in scavenging reactive oxygen species (ROS) and alleviating oxidative damage in laboratory studies (Mohanta, Sen, & Nayak, 2024). Research indicates that polysaccharides from *D. indica* also have immunomodulatory properties, enhancing phagocytosis and stimulating antibody production (Yeshwante et al., 2009). These features establish them as promising agents for natural immunotherapy and disease prevention. Studies suggest that they play a role in regulating gut health and improving digestive functions, which may benefit overall metabolic health (Rai & Sajwan, 2020).

### 4.8 Other bioactive compounds

**Fig. 10:**
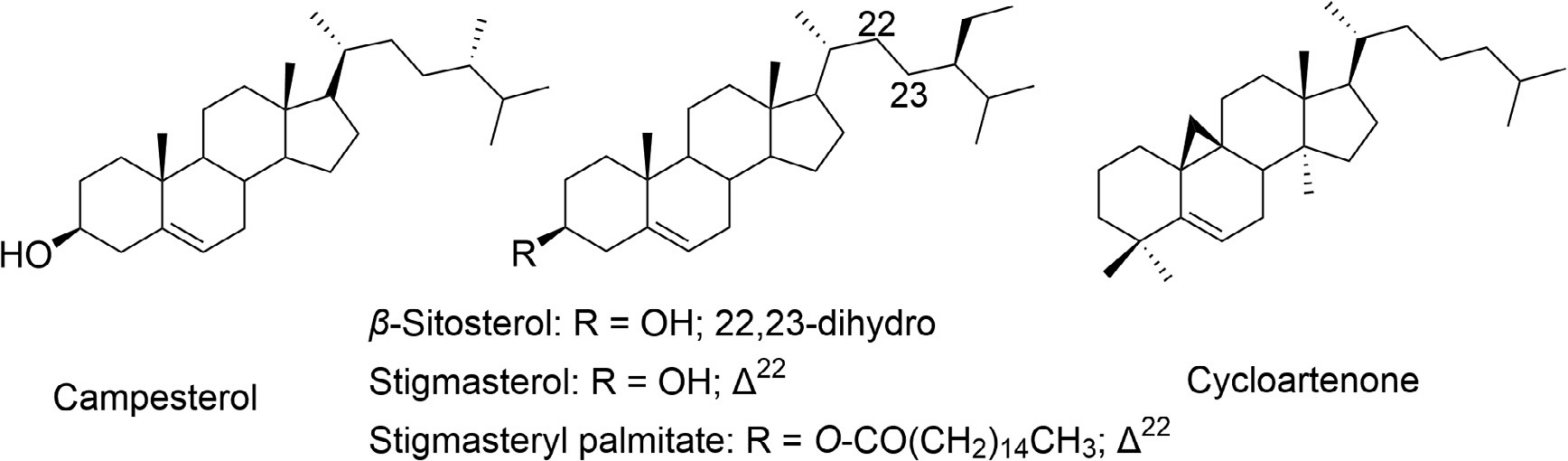
Bioactive sterols: Campesterol, stigmastrol, β-sitosterol, and cycloartenone from *D. indica.*

In addition to the major groups, *D. indica* contains several other bioactive compounds, including steroids and glycosides that enhance its therapeutic potential. The steroids present in this species contribute to anti-inflammatory processes by modulating immune responses, thereby reducing inflammation (Sabandar, Jalil, Ahmat, & Aladdin, 2017). Certain steroids, such as campesterol and *β*-sitosterol (Fig. 10), have been linked to cardiovascular benefits by improving lipid profiles and lowering cholesterol levels (Khan, Nath, Rauf, Bin Emran, Mitra, & Islam, 2022). Glycosides play significant roles in cardioprotective and antimicrobial activities. They work synergistically with triterpenoids and phenolic compounds to enhance the overall pharmacological profile of D. indica (Alam et al., 2020).

**Fig. 11:**
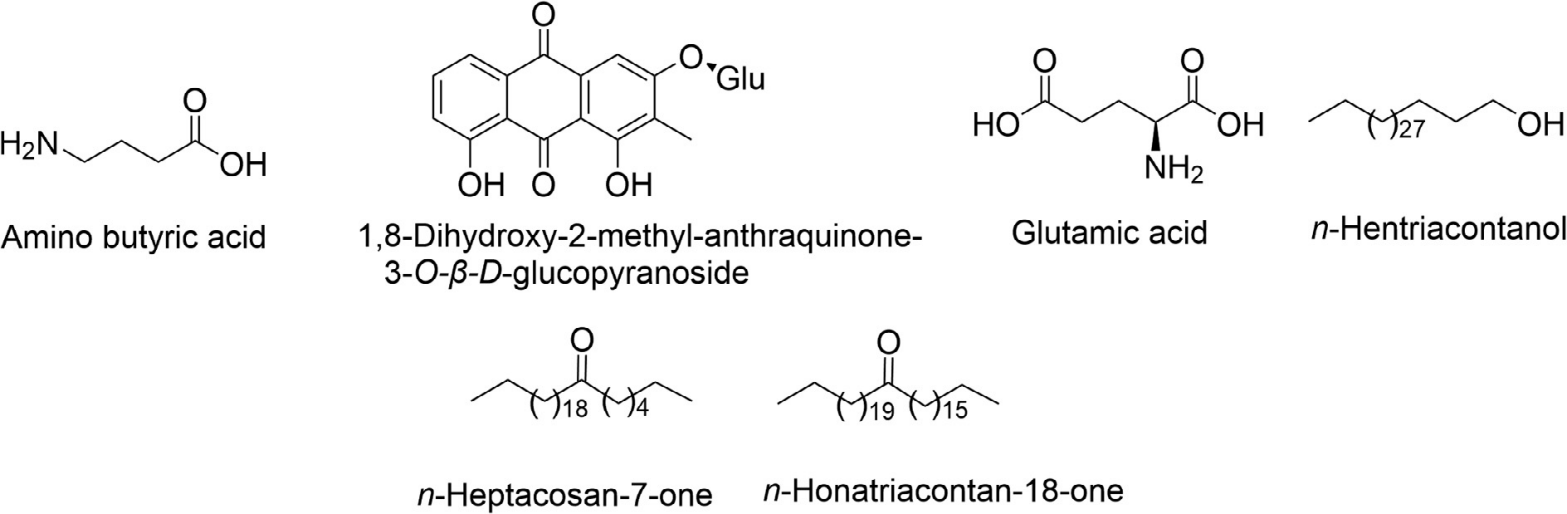
Miscellaneous compounds isolated from *D. indica*.

The presence of other bioactive compounds (Fig. 11), such as aminobutyric acid and glutamic acid, further supports the therapeutic versatility of *D. indica* (Saikia, Kesavan, Stephen Inbaraj, Dikkala, Nayak, & Sridhar, 2023).

## 5 Pharmacological and therapeutic potential of *D. indica*

*D. indica* is a medicinal plant with documented pharmacological properties. Traditional systems including Ayurveda and Siddha have employed this species for the treatment of various ailments. Recent studies have confirmed its therapeutic potential in managing oxidative stress, inflammation, microbial infections, cancer, and diabetes (Alam et al., 2012, Mohanta and Jena, 2023, Rai and Sajwan, 2020, Rizzo et al., 2020).

**Fig. 12:**
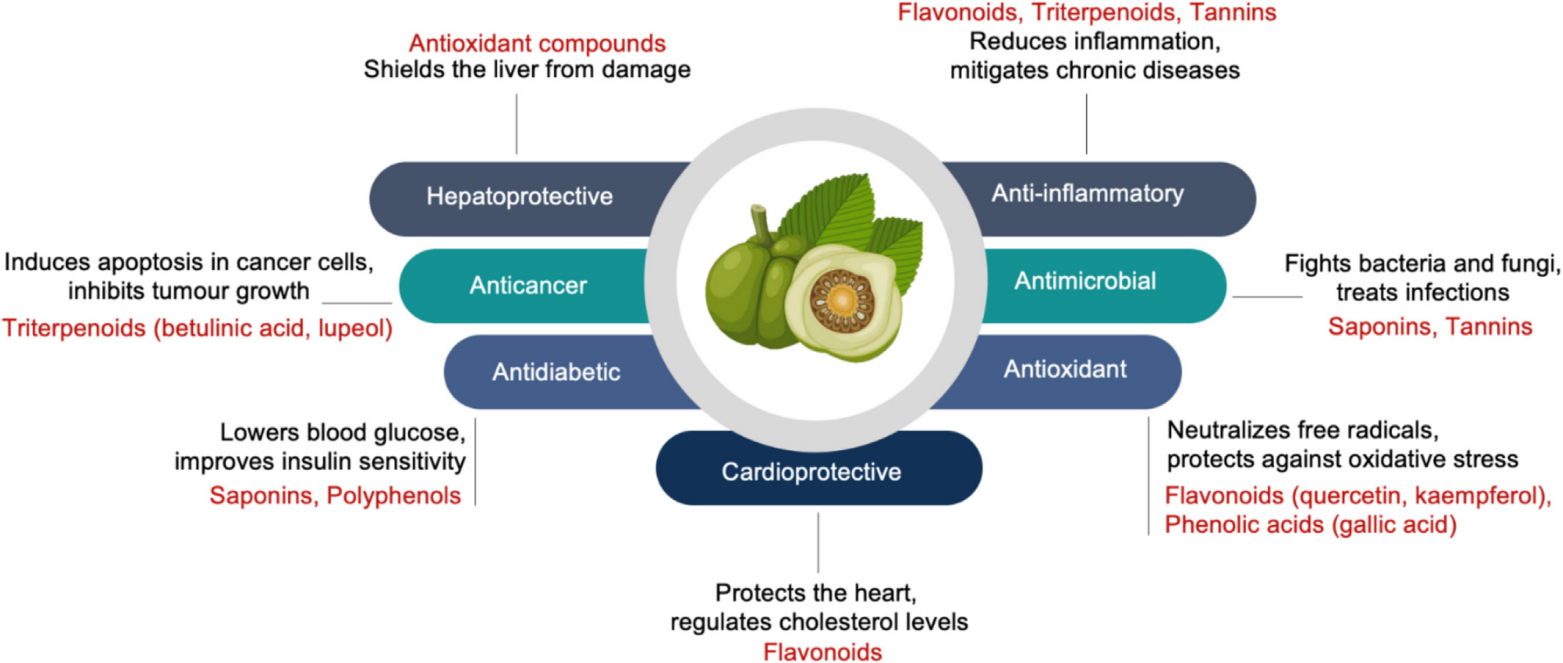
Pharmacological activities of *D. indica*.

The therapeutic efficacy of *D. indica* is attributed to its phytochemical constituents. These include flavonoids, tannins, triterpenoids, and polysaccharides (Yeshwante et al., 2009, Sharma et al., 2023). Flavonoids such as quercetin and kaempferol exhibit antioxidant and anti-inflammatory activities. These compounds neutralize reactive oxygen species and modulate immune responses (Kumar et al., 2011, Boparai et al., 2016). Moreover, synergistic interactions among these compounds enhance their therapeutic effects. Fig. 12 presents an overview of the pharmacological activities of *D. indica*.

### 5.1 Antioxidant activity

Oxidative stress contributes to the pathogenesis of cardiovascular disease, neurodegeneration, diabetes, and cancer. *D. indica* contains flavonoids and phenolic acids that exhibit strong antioxidant activity (Table 2). Quercetin, kaempferol, and gallic acid are the principal compounds responsible for this effect (Kaur et al., 2018, Alam et al., 2020, Mohanta and Jena, 2023).

**Table 2:** Antioxidant activity of bioactive compounds from *D. indica.*

Compounds	Assay types	IC_50_ (µg/mL)	Mechanisms of action	References
Quercetin	DPPH radical scavenging	15.6	Nrf2 pathway activation and ROS neutralization	Alam et al., 2020
Kaempferol	ABTS radical scavenging	20.3	Antioxidant enzyme activation; Superoxide dismutase (SOD) and catalase (CAT)	Kaur, Kishore, & Singh, 2018
Gallic acid	Hydroxyl radical assay	12.8	Free radical scavenging and metal chelation	Mohanta & Jena, 2023

Quercetin exhibits an half maximal inhibitory concentration (IC_50_) of 15.6 µg/mL in the DPPH assay and activates the Nrf2 pathway (Alam et al., 2020). Kaempferol exhibits an IC_50_ of 20.3 µg/mL in the ABTS assay and enhances superoxide dismutase and catalase activity (Kaur, Kishore, & Singh, 2018). Gallic acid exhibits an IC_50_ of 12.8 µg/mL in hydroxyl radical assay and contributes to metal ion chelation and free radical scavenging (Mohanta & Jena, 2023). Fruit polysaccharides also exhibit strong DPPH and ABTS radical scavenging activity, with IC_50_ values comparable to those of standard antioxidants (Mohanta, Sen, & Nayak, 2024).

### 5.2 Anticancer activity

Triterpenoids and flavonoids in *D. indica* exhibit cytotoxic effects against various cancer cell lines (Table 3). Betulinic acid induces apoptosis via mitochondrial pathways and activates Caspase-3 and Caspase-9 (Aswathy, Banik, Parama, Sasikumar, Harsha, & Joseph, 2021). Quercetin modulates phosphoinositide 3-kinase (PI3K)/protein kinase B (Akt) and mitogen-activated protein kinase (MAPK) signaling, inhibits proliferation, and promotes cell cycle arrest (Alam et al., 2020).

**Table 3:** Anticancer activity of bioactive compounds from *D. indica.*

Compounds	Target cell lines	IC_50_ (µg/mL)	Mechanisms of action	References
Betulinic acid	Oral squamous carcinoma cells	8.7	Mitochondrial apoptosis and caspase activation	Aswathy, Banik, Parama, Sasikumar, Harsha, & Joseph, 2021
Lupeol	Breast cancer cells	14.5	VEGF inhibition and PI3K-Akt modulation	Kaur, Kishore, & Singh, 2018
Quercetin	HeLa, A549, MCF-7	25 − 40	Cell cycle arrest and MAPK pathway inhibition	Alam et al., 2020

*In vitro* studies show IC_50_ values ranging from 25 − 40 µg/mL against HeLa, MCF-7, and A549 cells (Kaur, Kishore, & Singh, 2018). Lupeol inhibits vascular endothelial growth factor (VEGF) and modulates PI3K-Akt signaling (Kaur, Kishore, & Singh, 2018). *In vivo* models confirm tumor volume reduction without systemic toxicity (Padmavathi, Deshpande, & Sarala, 2011).

### 5.3 Antidiabetic activity

*D. indica* exhibits antidiabetic activity through inhibition of carbohydrate-hydrolyzing enzymes, enhancement of insulin sensitivity, and protection of pancreatic *β*-cells (Table 4). Gallic acid, quercetin, and betulinic acid are the principal compounds contributing to these effects (Mohanta and Jena, 2023, Padmavathi et al., 2011, Alam et al., 2020).

**Table 4:** Antidiabetic activity of bioactive compounds from *D. indica.*

Compounds	Assay/model	IC_50_/dose	Mechanisms of action	References
Gallic acid	*α*-Glucosidase inhibition	18.2 µg/mL	Enzyme inhibition and postprandial glucose control	Mohanta & Jena, 2023
Gallic acid	*α*-Amylase inhibition	22.5 µg/mL	Suppression of starch digestion	Mohanta & Jena, 2023
Quercetin	Streptozotocin (STZ)-induced diabetic rats	200 mg/kg	AMPK activation and insulin sensitization	Padmavathi, Deshpande, & Sarala, 2011
Betulinic acid	*In vivo* diabetic model	Not reported	*β*-cell protection and insulin enhancement	Alam et al., 2020

Gallic acid inhibits *α*-glucosidase and *α*-amylase as carbohydrate-hydrolyzing enzymes with IC_50_ values of 18.2 µg/mL and 22.5 µg/mL, respectively (Mohanta & Jena, 2023). Quercetin activates AMP-activated protein kinase and improves glucose uptake in peripheral tissues and causes enhancement of insulin sensitivity (Padmavathi, Deshpande, & Sarala, 2011). Betulinic acid enhances insulin secretion and protects pancreatic *β*-cells from oxidative damage (Alam et al., 2020). In streptozotocin-induced diabetic rats, *D. indica* extract at 200 mg/kg reduces fasting blood glucose by 35 %−45 % over 21 days (Padmavathi, Deshpande, & Sarala, 2011).

### 5.4 Anti-inflammatory activity

Flavonoids, triterpenoids, and phenolic acids in *D. indica* inhibit inflammatory mediators and enzymes (Table 5). Quercetin suppresses nuclear factor-*κ*B (NF-*κ*B) activation and downregulates tumor necrosis factor-*α* (TNF-*α*), IL-1*β*, and IL-6 (Alam et al., 2020). Betulinic acid inhibits cyclooxygenase-2 (COX-2) and inducible nitric oxide synthase (iNOS), reducing prostaglandin and nitric oxide levels (Mohanta & Jena, 2023).

**Table 5:** Anti-inflammatory activity of bioactive compounds from *D. indica.*

Compounds	Assay/model	IC_50_/dose	Mechanisms of action	References
Quercetin	NF-*κ*B inhibition assay	Not reported	Suppression of TNF-*α* and IL-6	Alam et al., 2020
Betulinic acid	COX-2 and iNOS inhibition	Not reported	Reduction of prostaglandin and nitric oxide	Mohanta & Jena, 2023
Methanolic extract	Carrageenan-induced paw edema	250 mg/kg	Edema reduction and leukocyte inhibition	Padmavathi, Deshpande, & Sarala, 2011
Mixed flavonoids	5-LOX and COX enzyme inhibition	16.7 − 21.3 µg/mL	Enzymatic suppression	Mohanta & Jena, 2023

In carrageenan-induced paw edema models, methanolic extract at 250 mg/kg reduces inflammation by 48 %−55 %, comparable to diclofenac (Padmavathi, Deshpande, & Sarala, 2011). Additionally, mixed flavonoids inhibit 5-lipoxygenase (5-LOX) and COX enzymes with IC_50_ values ranging from 16.7 − 21.3 µg/mL (Mohanta & Jena, 2023). Histological analysis confirms reduced leukocyte infiltration and edema.

### 5.5 Antimicrobial and antiviral activity

*D. indica* exhibits broad-spectrum antimicrobial and antiviral activity (Table 6). Methanolic extracts inhibit Gram-positive and Gram-negative bacteria including *S. aureus* and *E. coli*, with minimum inhibitory concentration values of 62.5 µg/mL (Alam et al., 2012, Rai and Sajwan, 2020, Rizzo et al., 2020). Tannins show antifungal activity against *Candida albicans* with MIC of 50.0 µg/mL (Boparai, Niazi, Bajwa, & Singh, 2016). Flavonoid-rich extracts inhibit *Pseudomonas aeruginosa* and *Aspergillus niger* with MIC values ranging from 75 − 90 µg/mL (Padmavathi, Deshpande, & Sarala, 2011).

**Table 6:** Antimicrobial and antiviral activity of bioactive compounds from *D. indica.*

Compounds/extracts	Targets/models	MIC/EC_50_ (µg/mL)	Mechanisms of action	References
Methanolic extract	*S. aureus*; *E. coli*	62.5	Membrane disruption and efflux pump inhibition	Alam et al., 2020, Rai and Sajwan, 2020, Rizzo et al., 2020
Tannins	*C. albicans*	50	Cell wall interference and antifungal activity	Boparai, Niazi, Bajwa, & Singh, 2016
Mixed extract	HSV-1; Influenza A virus	32.4 − 45.1	Inhibition of viral entry and replication	Mohanta & Jena, 2023
Flavonoid-rich extract	*P. aeruginosa, A. niger*	75 − 90	Quorum sensing and biofilm inhibition	Padmavathi, Deshpande, & Sarala, 2011

Mixed extracts of *D. indica*, comprising flavonoids, tannins, and triterpenoids, have exhibited significant antiviral activity against herpes simplex virus type 1 and influenza A virus, with EC_50_ values ranging from 32.4 − 45.1 µg/mL (Mohanta & Jena, 2023). The observed effects are mediated through a combination of mechanisms, including disruption of viral membranes, inhibition of DNA gyrase, and suppression of viral entry and intracellular replication.

## 6 Preclinical and clinical studies

The pharmacological properties of *D. indica* have been extensively studied through preclinical research, including both *in vitro* experiments and *in vivo* animal models (Alam et al., 2012, Rai and Sajwan, 2020, Rizzo et al., 2020, Sharma et al., 2023). These studies demonstrate a wide range of therapeutic benefits linked to this plant, particularly its antioxidant, anti-inflammatory, antimicrobial, anticancer, and antidiabetic effects (Kamboj et al., 2019, Mohanta and Jena, 2023). These findings provide a strong foundation for potential clinical applications (Boparai et al., 2016, Rizzo et al., 2020). A summary of preclinical and clinical findings on *D. indica* is provided in Table 7. Further investigation into its bioactive compounds and mechanisms of action is essential for fully realizing the therapeutic potential of this plant and for integrating these natural remedies into modern medicine.

**Table 7:** Summary of preclinical and clinical findings on *D. indica*.

Study types	Bioactivities	Bioactive compounds	Key findings	References
Preclinical *in vitro*	Anticancer	Betulinic acid; Lupeol	Induction of apoptosis in cancer cells and demonstrates low IC_50_ values against various cancer lines	Kumar et al., 2010, Aswathy et al., 2021
Preclinical *in vitro*	Anti-inflammatory	Flavonoids; Triterpenoids	Inhibition of COX-2 and reduction of pro-inflammatory cytokine levels	Kamboj et al., 2019, Alam et al., 2020
Preclinical *in vitro*	Antimicrobial	Saponins; Tannins	Significant antibacterial and antifungal activity with MIC values from 50 to 100 µg/mL	Alam et al., 2012, Boparai et al., 2016
Preclinical *in vitro*	Antioxidant	Flavonoids (Quercetin; Kaempferol)	Exhibition of strong free radical scavenging activity and enhancement of oxidative stress management	Kamboj et al., 2019, Mohanta and Jena, 2023
Preclinical *in vivo*	Antidiabetic	Saponins; Polyphenols	Decrease of blood glucose levels and improvement of insulin sensitivity	Kamboj et al., 2019, Mohanta and Jena, 2023
Preclinical *in vivo*	Antimicrobial	Saponins; Tannins	Enhancement of wound healing and reduction of bacterial load in infected wounds	Boparai et al., 2016, Mohanta and Jena, 2023
Preclinical *in vivo*	Cardioprotective	Flavonoids	Protection against vascular injury and inflammation	Kamboj et al., 2019, Singh and Saha, 2019
Preclinical *in vivo*	Hepatoprotective	Antioxidant compounds	Protection of liver function and reduction of alanine aminotransferase (ALT) and aspartate aminotransferase (AST) levels	Kaur et al., 2018, Rizzo et al., 2020
Clinical observations	Antidiabetic	Flavonoids; Tannins	Evidence of improving blood glucose levels in patients	Kamboj et al., 2019, Boruah et al., 2020

### 6.1 Preclinical *in vitro* studies

#### 6.1.1 Anticancer effects

Research highlights the anticancer potential of *D. indica*. The plant contains bioactive compounds, particularly triterpenoids like betulinic acid and lupeol, which have been shown to induce apoptosis in cancer cells (Aswathy et al., 2021, Sahariah et al., 2023). Recent studies indicate that extracts from *D. indica* can significantly reduce the viability of various cancer cell lines, including oral squamous cell carcinoma and breast cancer cells (Kaur et al., 2018, Lawal et al., 2021). The anticancer mechanisms include inhibiting angiogenesis through the downregulation of VEGF (Kamboj et al., 2019, Singh and Saha, 2019).

It’s well-known that flavonoids in *D. indica* help mitigate oxidative stress and inflammation, enhancing its anticancer efficacy (Alam et al., 2020). The combined action of these phytochemicals suggests a multifaceted approach to cancer treatment, warranting further exploration in oncology. While preclinical studies demonstrate significant potential, comprehensive human clinical trials are crucial to validate the efficacy and safety of *D. indica* for therapeutic use (Sabandar, Jalil, Ahmat, & Aladdin, 2017). The standardization of extraction methods is essential for reproducibility. Further research should focus on identifying novel bioactive compounds, elucidating the mechanisms of action, and investigating potential drug interactions. The development of advanced drug delivery systems may improve therapeutic efficacy (Sarker & Nahar, 2012).

#### 6.1.2 Anti-inflammatory effects

The anti-inflammatory effects of *D. indica* are linked to its flavonoids, triterpenoids, and tannins (Alam et al., 2012, Kamboj et al., 2019). These compounds modulate inflammatory pathways by inhibiting COX-2 and lowering pro-inflammatory cytokines, including TNF-*α* and IL-6 (Rai and Sajwan, 2020, Mohanta and Jena, 2023). D. indica extracts significantly reduce prostaglandin synthesis, suggesting potential in managing chronic inflammatory conditions such as arthritis and inflammatory bowel disease (Boparai et al., 2016, Sharma et al., 2023).

#### 6.1.3 Antimicrobial effects

The antimicrobial properties of *D. indica* are well-documented, demonstrating efficacy against bacterial and fungal pathogens. Methanolic extracts have exhibited significant antibacterial activity against *S. aureus* and *E. coli,* with minimum inhibitory concentration (MIC) values recorded at 62.5 µg/mL (Alam et al., 2012, Mohanta and Jena, 2023). Extracts also show antifungal effects against *C. albicans* and *A. niger* (Boparai et al., 2016, Kamboj et al., 2019). The antimicrobial action involves disrupting microbial cell membranes and inhibiting biofilm formation, reinforcing its traditional use in treating infections.

#### 6.1.4 Antioxidant effects

*D. indica* is a potent antioxidant due to its high flavonoid and phenolic acid content (Alam et al., 2012, Kamboj et al., 2019). These compounds neutralize reactive oxygen species (ROS), reducing oxidative stress, which is a key factor in many chronic diseases. Studies confirm that *D. indica* extracts significantly lower oxidative stress markers and enhance antioxidant enzyme activity, protecting against oxidative damage linked to chronic diseases (Rai and Sajwan, 2020, Mohanta and Jena, 2023).

### 6.2 Preclinical *in vivo* studies

#### 6.2.1 Anticancer effects

*In vivo* studies using cancer models confirm the anticancer properties of *D. indica*. Treatment in mice with *Ehrlich ascites* carcinoma results in significant reductions in tumour volume and weight, indicating potent anticancer activity (Kumar et al., 2010, Aswathy et al., 2021). The extracts induce apoptosis and inhibit angiogenesis, reinforcing their therapeutic potential (Lawal et al., 2021, Mohanta and Jena, 2023).

#### 6.2.2 Antidiabetic effects

Recent studies focus on the antidiabetic properties of *D. indica*. *In vivo* research demonstrates that *D. indica* extracts inhibit carbohydrate-digesting enzymes, such as alpha-glucosidase and alpha-amylase, aiding in blood glucose regulation (Kamboj, Talukdar, & Banerjee, 2019). Additionally, flavonoids and saponins in *D. indica* protect pancreatic beta cells from oxidative stress, further supporting its antidiabetic potential (Kaur et al., 2018, Sen et al., 2018, Mohanta and Jena, 2023).

#### 6.2.3 Anti-inflammatory effects

The anti-inflammatory properties of *D. indica* are demonstrated in studies involving oral administration in animal models. These studies show significant reductions in inflammatory responses, such as paw oedema (Alam et al., 2012, Kamboj et al., 2019). The compounds in *D. indica* modulate inflammatory pathways, inhibiting COX-2 and lowering pro-inflammatory cytokines, indicating potential for therapeutic applications in chronic inflammatory conditions (Rai and Sajwan, 2020, Mohanta and Jena, 2023).

#### 6.2.4 Antimicrobial effects

*In vivo* studies validate the antimicrobial efficacy of *D. indica*. Experiments involving infected wounds in rats demonstrate the plant's efficacy against bacterial and fungal pathogens. Methanolic extracts show significant antibacterial activity against *S. aureus* and *E. coli*, with MIC values recorded at 62.5 µg/mL (Alam et al., 2012, Mohanta and Jena, 2023). *D. indica* extracts exhibit antifungal effects against *C. albicans* and *A. niger* (Boparai et al., 2016, Kamboj et al., 2019). The antimicrobial action involves disrupting microbial cell membranes and inhibiting biofilm formation, reinforcing its traditional use in treating infections.

#### 6.2.5 Antioxidant effects

*In vivo* studies confirm the antioxidant findings from *in vitro* research, it is a potent antioxidant due to its high flavonoid and phenolic acid content (Alam et al., 2012, Kamboj et al., 2019). Research confirms that *D. indica* extracts significantly lower oxidative stress markers and enhance antioxidant enzyme activity, protecting against oxidative damage linked to chronic diseases (Rai and Sajwan, 2020, Mohanta and Jena, 2023).

Recent investigations have also highlighted the antioxidant efficacy of fruit-derived polysaccharides. These high-molecular-weight compounds significantly reduce oxidative damage and support systemic antioxidant defense mechanisms in animal models (Mohanta, Sen, & Nayak, 2024). The polysaccharides exhibit strong DPPH and ABTS radical scavenging activity and show excellent biocompatibility, reinforcing their potential for safe therapeutic use.

Together, these findings support the role of *D. indica* as a natural antioxidant source with broad therapeutic relevance, particularly in the prevention and management of oxidative stress-related disorders.

### 6.3 Critical evaluation of clinical observations and translational challenges

Clinical studies suggest that *D. indica* has a beneficial role in managing diabetes. Studies indicate that patients consuming *D. indica* extracts experience significant reductions in blood glucose levels and improvements in insulin sensitivity. The presence of bioactive compounds, such as flavonoids and tannins, contributes to these effects by enhancing glucose metabolism and reducing oxidative stress (Kamboj et al., 2019, Boruah et al., 2020). However, the available clinical data remain preliminary and lack scientific robustness. Most studies are limited to small-scale, non-randomized trials with short durations and limited participant diversity. Furthermore, these studies often do not report precise information regarding extract composition, dosage standardization, or adverse effects monitoring, making reproducibility and generalization difficult.

There is also a lack of comprehensive pharmacokinetic and pharmacodynamic data in humans. Key factors such as bioavailability, metabolism of active phytoconstituents (e.g., betulinic acid, quercetin), and potential interactions with other medications remain uncharacterized. The absence of placebo-controlled, double-blinded trials is a major limitation that prevents drawing strong conclusions about efficacy. This disparity between robust preclinical evidence including anticancer, antidiabetic, and anti-inflammatory activity, and weak clinical validation highlights the urgent need for well-designed randomized controlled trials (RCTs). These trials should incorporate defined extract preparations, dosage optimization, long-term safety assessments, and population diversity to bridge the translational gap and enable future therapeutic applications.

## 7 Future directions and research gaps

Despite encouraging findings from preclinical studies, considerable research gaps exist that must be addressed to advance the clinical application of *D. indica*. One major challenge involves the absence of well-designed human clinical trials, which are necessary to validate the promising results observed in preclinical studies. While numerous studies highlight the potential benefits of *D. indica,* yet further human research is essential to confirm these effects (Sabandar et al., 2017, Kamboj et al., 2019, Mohanta and Jena, 2023, Sharma et al., 2023).

### 7.1 Clinical trials and human studies

The conduction of clinical trials is vital for determining optimal dosages and evaluating the bioavailability of extracts from *D. indica*. The identification of potential side effects of these extracts is also critical. RCTs utilizing standardized extracts are necessary for translating promising preclinical findings into practical clinical applications. Such trials will establish efficacy and safety across various health conditions, and more reliable results will ensure applicability to broader patient populations (Boparai et al., 2016, Rizzo et al., 2020).

### 7.2 Standardization of extracts

A significant issue presents itself in the form of limited standardized protocols for extracting bioactive compounds. Variation in compound content is influenced by multiple factors, including growth conditions, harvesting time, and extraction methods (Kaur, Kishore, & Singh, 2018). This inconsistency complicates the reproducibility of therapeutic outcomes. Future research should prioritize the development of standardized extraction protocols. The establishment of consistent concentrations of key bioactive compounds, such as flavonoids and triterpenoids, is essential (Boruah, Saikia, Islam, Das Purkayastha, & Borah, 2020). Additionally, the isolation and identification of specific compounds responsible for therapeutic effects may lead to more targeted treatments (Sharma, Sharma, & Koul, 2023).

### 7.3 Mechanistic insights, pharmacodynamics, and future perspectives

**Fig. 13:**
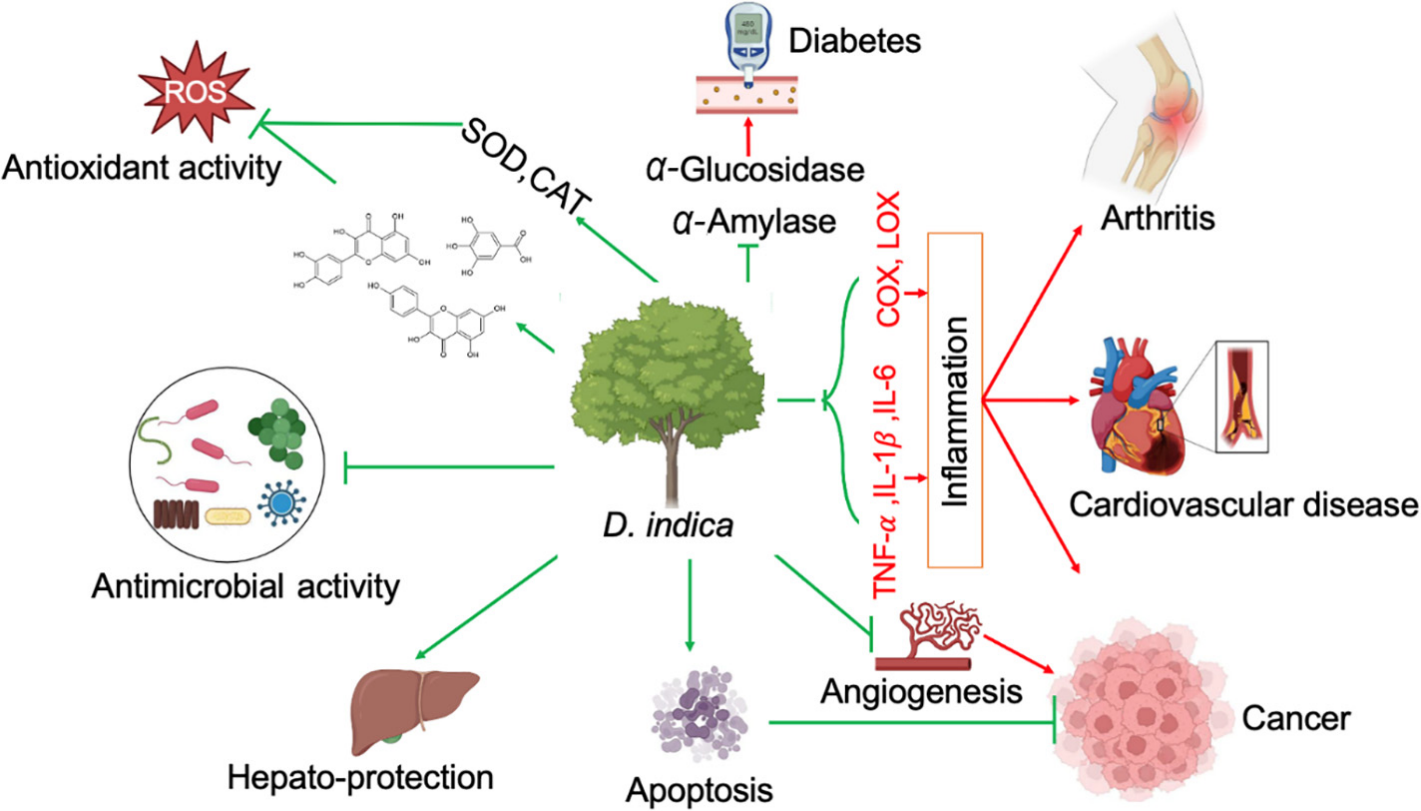
Action modes of *D. indica* in various therapeutic applications.

*D. indica* exhibits a broad spectrum of pharmacological activities, including antioxidant, anti-inflammatory, anticancer, and antimicrobial effects (Fig. 13). These activities have been attributed to specific phytochemicals such as quercetin, kaempferol, betulinic acid, and saponins (Kamboj et al., 2019, Kaur et al., 2018, Padmavathi et al., 2011, Lawal et al., 2021). However, most findings are based on *in vitro* assays and general biochemical observations. The molecular mechanisms responsible for these effects remain poorly defined. For example, triterpenoids such as betulinic acid have demonstrated the ability to induce apoptosis and suppress angiogenesis in cancer cells (Aswathy et al., 2021), yet the intracellular signaling pathways involved in these actions have not been clearly characterized.

Advanced mechanistic studies are needed to identify the molecular targets and pathways affected by *D. indica* compounds. Techniques such as transcriptomics, proteomics, and molecular docking should be employed to investigate compound-specific actions. Integration of these approaches with *in vivo* pharmacodynamic modeling will provide a more complete understanding of therapeutic mechanisms. In addition, the interactions between multiple bioactive compounds within *D. indica* remain insufficiently studied. While quercetin and kaempferol are both known antioxidants, their combined influence on signaling pathways such as Nrf2/HO-1, PI3K/Akt, and NF-κB has not been evaluated in physiological models (Kaur et al., 2018, Padmavathi et al., 2011).

To address these gaps, future research should focus on five key areas:

i. Elucidating compound-specific signaling pathways using omics technologies and clustered regularly interspaced short palindromic repeats (CRISPR)-based functional studies.
ii. Conducting systematic pharmacokinetic (PK) and pharmacodynamic (PD) profiling in animal models and human trials.
iii. Investigating compound synergy through combination studies and network pharmacology.
iv. Standardizing extracts to ensure reproducibility and dose–response consistency.
v. Applying computational modeling, including molecular dynamics simulations and artificial intelligence (AI)-based docking, to predict interactions with molecular targets.

These efforts will be critical for validating *D. indica* as a reliable therapeutic agent and for supporting its integration into modern drug development pipelines.

### 7.4 Drug interactions and pharmacokinetics

Current pharmacokinetic studies on *D. indica* are limited. This limitation hinders a comprehensive understanding of the absorption, distribution, metabolism, and excretion (ADME) profiles of the plant (Mohanta & Jena, 2023). The lack of extensive research poses risks, particularly when *D. indica* is combined with conventional drugs metabolized by cytochrome P450 (CYP450) enzymes. Future studies need to investigation into these drug interactions and the evaluation of the bioavailability of active constituents in oral formulations. This research is essential for establishing safe dosage guidelines and minimizing adverse effects, especially for individuals with chronic conditions or those on long-term medication (Kamboj et al., 2019, Mohanta and Jena, 2023).

### 7.5 Toxicology and long-term safety

Preclinical models indicate a favourable toxicological profile for *D. indica*. However, further research is essential for evaluating its long-term safety. This evaluation is particularly important at higher doses or with prolonged use (Alam et al., 2012). Chronic toxicity studies should focus on the cumulative effects of *D. indica* on vital organs, particularly the liver and kidneys. An assessment of any potential long-term impacts associated with extended consumption is critical (Mohanta & Jena, 2023). The evaluation of reproductive and developmental toxicity remains crucial. It is important to ensure the safety of *D. indica* for pregnant and breastfeeding women. The findings in these areas will provide a comprehensive understanding of the safety profile of *D. indica* within various populations (Sharma, Sharma, & Koul, 2023).

Recent findings have confirmed that fruit-derived polysaccharide extracts are non-toxic in acute models, with no adverse effects observed in mice up to 1 500 mg/kg (Mohanta, Sen, & Nayak, 2024). These results support the systemic safety of fruit-based formulations. In addition, bark-derived copper nanoparticles have demonstrated selective cytotoxicity against cancer cells without inducing systemic toxicity in normal tissues, reinforcing their therapeutic potential (Mohanta & Jena, 2023). Together, these findings suggest that *D. indica* possesses a favourable safety margin, but comprehensive long-term and reproductive toxicity studies are required to establish its suitability for broader clinical use.

### 7.6 Formulation development and advanced delivery systems

Formulation strategies have the potential to significantly enhance the therapeutic effects of *D. indica*. Modern pharmaceutical technologies, such as encapsulation, nanoformulations, and sustained-release systems, can substantially improve the solubility and stability of the bioactive compounds contained within the plant (Sabandar, Jalil, Ahmat, & Aladdin, 2017). These advanced delivery systems play a vital role in increasing therapeutic efficacy, particularly in the treatment of cancer, inflammation, and oxidative stress-related diseases (Rai & Sajwan, 2020). Techniques such as these protect sensitive compounds from degradation while enhancing absorption in the gastrointestinal tract. The delivery of bioactive compounds to specific target tissues is improved through nanoformulations, maximizing their pharmacological effects.

The incorporation of *D. indica* extracts into pharmaceutical formulations, such as gels, emulsions, and tablets, can broaden the applications of this plant beyond its traditional uses. Mucoadhesive formulations leverage the mucilage properties of *D. indica*, enhancing drug retention in the nasal cavity. This method may improve delivery to the central nervous system (Kaur, Kishore, & Singh, 2018). Future research should focus on optimizing these delivery systems, including the evaluation of the physicochemical properties of the formulations and the assessment of the release profiles of the bioactive compounds. Furthermore, examination of their pharmacokinetic behaviours is vital. Such efforts will contribute to the development of safe, effective, and user-friendly therapeutic options based on *D. indica*.

### 7.7 Identification of other unknown bioactive compounds

Current research primarily emphasizes known compounds, such as flavonoids and triterpenoids. However, significant potential remains for *D. indica* to contain additional unidentified bioactive components with therapeutic potential (Kamboj et al., 2019, Mohanta and Jena, 2023). Advances in phytochemical analysis techniques provide valuable opportunities for discovering and characterizing these new compounds. Analytical techniques such as high-performance liquid chromatography (HPLC), mass spectrometry (MS), and nuclear magnetic resonance (NMR) spectroscopy can play a significant role in this research (Sarker & Nahar, 2012).

Recent studies have emphasized the immunomodulatory and antioxidant effects of isolated polysaccharides from *D. indica*, highlighting the need for further exploration of the chemical profile of *D. indica* (Alam et al., 2020, Mohanta et al., 2024). The identification of these unknown bioactive compounds may yield novel therapeutic applications and enhance the overall utility of *D. indica* in modern medicine. A thorough phytochemical investigation is essential for future research to uncover these compounds. This extensive profiling not only validates traditional medicinal practices but also paves the way for new developments in drug formulation. Such advancements could be particularly beneficial in various fields, including oncology, diabetes management, and chronic disease prevention (Padmavathi et al., 2011, Nagar et al., 2020). By exploring the potential of *D. indica*, researchers can contribute to the integration of traditional knowledge with contemporary scientific approaches, fostering innovative health solutions.

### 7.8 Preservation of sustainable cultivation

The increasing demand for *D. indica* due to its medicinal and culinary uses makes the establishment of sustainable cultivation practices essential (Mohanta, Sen, & Nayak, 2024). Such practices play a critical role in preventing overharvesting and ensuring the preservation of genetic diversity within the species (Rai & Sajwan, 2020). Bark harvesting, particularly for nanoparticle synthesis, must be carefully managed to avoid ecological degradation and ensure long-term resource availability (Mohanta & Jena, 2023).

Future research should focus on optimizing cultivation conditions, including soil types, climate, and farming techniques to maximize yield and quality. Controlled agricultural methods are necessary to prevent ecosystem degradation (Boruah, Saikia, Islam, Das Purkayastha, & Borah, 2020). An integrated approach combining ecological preservation with ongoing pharmacological research will enhance the viability of *D. indica* as a long-term resource for therapeutic development (Mohanta, Sen, & Nayak, 2024).

The implementation of robust conservation strategies will be vital to ensuring the long-term availability of this valuable plant for both culinary and medicinal purposes. Educating for local farmers regarding sustainable harvesting methods is also important. The community-based conservation efforts can help maintain ecological balance while supporting the livelihoods of those engaged in *D. indica* cultivation. An integrated approach that combines sustainable agricultural practices with ongoing research into the properties of the plant can significantly enhance the viability of *D. indica* as a resource for future medicinal applications.

### 7.9 Comparative analysis with other medicinal plants

While many plant species have been widely studied for their medicinal properties, *D. indica* exhibits a distinctive profile both phytochemically and pharmacologically. Unlike *Curcuma longa* L., which is rich in curcuminoids, or *Azadirachta indica* A. Juss., known for its limonoids and potent antimicrobial activity, *D. indica* offers a unique combination of triterpenoids (e.g., betulinic acid, lupeol), flavonoids (e.g., quercetin, kaempferol), and polysaccharides with immunomodulatory effects (Kaur et al., 2018, Mohanta and Jena, 2023, Mohanta et al., 2024). Its simultaneous antioxidant, anti-inflammatory, antidiabetic, and anticancer properties suggest synergistic therapeutic potential.

In contrast to *Phyllanthus emblica* L., which is primarily studied for its vitamin C content and antioxidant properties, *D. indica* combines antioxidant efficacy with strong antimicrobial and anticancer activities supported by diverse phytoconstituents, including saponins, tannins, and fruit-derived polysaccharides (Alam et al., 2020, Mohanta et al., 2024).

Moreover, while *C. longa* has progressed toward standardized formulations and clinical trials, *D. indica* remains underexplored in terms of clinical translation, despite its promising pharmacological profile. Although widely used in traditional medicine across Northeast India, only limited clinical investigations have been conducted to validate its therapeutic efficacy, particularly in metabolic disorders such as diabetes (Kamboj, Talukdar, & Banerjee, 2019). This gap highlights a valuable opportunity for future pharmaceutical development.

Overall, *D. indica* may serve as a complementary or alternative natural therapy, especially where multi-targeted mechanisms are required, such as in chronic inflammation or metabolic syndrome. Its comparative strengths lie in its broad-spectrum phytochemical diversity, including triterpenoids, flavonoids, and polysaccharides, and its integration into food-medicine systems, which support its potential as a functional botanical (Saiful Yazan and Armania, 2014, Kamboj et al., 2019).

## 8 Conclusion

*D. indica* shows significant promise as a medicinal plant with a rich history in traditional medicine throughout Asia. The therapeutic potential of *D. indica* is attributed to its variety of bioactive compounds, which include flavonoids, triterpenoids, tannins, saponins, and polysaccharides. These compounds contribute to a wide range of pharmacological activities, demonstrating effects such as antioxidant, anti-inflammatory, antimicrobial, anticancer, and antidiabetic properties. Modern scientific research has begun to support these traditional uses, especially for diseases related to oxidative stress, chronic inflammation, infections, diabetes, and cancer. Despite these encouraging findings, significant research gaps remain. More human clinical trials are essential to fully understand the therapeutic potential and safety profile of *D. indica*. Standardization of its extracts is critical for ensuring consistent therapeutic outcomes. Detailed mechanistic studies are also necessary to clarify the molecular pathways behind its effects. The development of advanced drug delivery systems is important for enhancing the pharmacological benefits of *D. indica*. Additional toxicological and pharmacokinetic assessments are needed to establish optimal dosages and understand potential interactions with conventional medications. *D. indica* presents a unique opportunity to bridge traditional knowledge with modern medicine. It may provide safe and effective natural alternatives for managing various health conditions.

## CRediT authorship contribution statement

**Lutfun Nahar:** Conceptualization, Visualization, Validation, Project administration, Writing – original draft, Writing – review & editing. **Emran Habibi:** Software, Writing – original draft, Writing – review & editing. **Chuanchom Khuniad:** Writing – original draft, Writing – review & editing. **Kulyash Kalieva:** Writing – original draft, Writing – review & editing. **Daijie Wang:** Writing – original draft, Writing – review & editing. **Hesamoddin Arabnozari:** Writing – original draft, Writing – review & editing. **Phanuphong Chaiwut:** Writing – original draft, Writing – review & editing. **Sarita Sangthong:** Writing – original draft, Writing – review & editing. **Tinnakorn Theansungnoen:** Writing – original draft, Writing – review & editing. **Rajat Nath:** Writing – original draft, Writing – review & editing. **Anupam Das Talukdar:** Writing – original draft, Writing – review & editing. **Satyajit D. Sarker:** Writing – original draft, Writing – review & editing.

## Declaration of Competing Interest

The authors declare that they have no known competing financial interests or personal relationships that could have appeared to influence the work reported in this paper.
